# Treatment of Adults with Anterior Mandibular Teeth Crowding: Reliability of Little's Irregularity Index

**DOI:** 10.1155/2017/5057941

**Published:** 2017-02-06

**Authors:** J. Antoszewska-Smith, M. Bohater, M. Kawala, M. Sarul, M. Rzepecka-Skupień

**Affiliations:** ^1^Department of Dentofacial Orthopedics and Orthodontics, Wroclaw Medical University, Wroclaw, Poland; ^2^Department of Prosthodontics, Wroclaw Medical University, Wroclaw, Poland

## Abstract

The attempt of this article was to assess reliability of Little's Irregularity Index (LII) as for stability of the treatment outcomes in adults with crowded mandibular incisors. LII was measured on a digital cast prior to an orthodontic treatment (T1) of the 302 patients thus allowing us to establish the treatment plan, which called for (a) expansion (group 1), interproximal stripping (group 2), or extraction of one of the mandibular incisors. LII was measured after debonding (T2) and a year after retention (T3). Treatment resulted in significant reduction of LII values after treatment, in T1-T2 period in all groups. As for T2-T3 period it brought significant but clinically irrelevant relapse that occurred in groups 1 and 2; group 3 presented with insignificant improvement of occlusion. Conclusively, 30 years after introducing LII it has been a reliable parameter that allows selection of optimal treatment methods, provided that the appropriate ranges of values displaying dentoalveolar discrepancy are obeyed, namely, (1) up to 3 mm: expansion, (2) from 3 to 5 mm: interproximal enamel reduction, and (3) above 5 mm: extraction.

## 1. Introduction

Nowadays orthodontics not only is the treatment of children and adolescents: booming development of the therapeutic techniques has excluded an age from the list of limiting factors. Therefore, the number of adults actively seeking help to correct their malocclusion has been constantly increasing, possibly due to the social reasons [1–3]. The most common abnormality they present with is crowding of the lower incisors, of which etiology and prevention have been fervently discussed for years.

Studies from the past three decades brought the evidence that individuals over twenty mostly exhibit late crowding [4–6]. Previously, Begg [7] and Müller [8] attempted to elucidate its etiology. Analyzing prehistoric populations Begg found that insufficient interproximal and occlusal enamel reduction may be a factor, whereas Müller suggested that cheek overpressure developed due to improperly lowered tongue posture is likely to tilt the teeth, which reduces space they normally occupy. In turn Masztalerz [9] as well as Corruccini [10] associated crowding with gradual reduction of the craniofacial structures rather than with the teeth size. On the contrary Bishara et al. [11] and Vaden et al. [12] proved that mandibular growth that does not cease after puberty but continues, at a slower rate, throughout adulthood independently on the patients' gender might be a cause. Increase of the mandibular length reduces an overjet and, subsequently, space for the lower incisors exposing them to crowding. Last but not least mesial movement of third molars is the most controversial concept: Nanda [13], Southard et al. [14], and Mockers et al. [15] found no evidence on the contrary to Mockers et al. [16], Šidlaukas and Trakinienê [17], and Tüfekçi et al. [18] who confirmed the discussed negative impact of third molars thus establishing their extraction as the prophylaxis of late crowding. As etiology of the mandibular anterior teeth has been an obvious matter of dispute, the mode of an orthodontic treatment in adults who do not accept extraction of two or four premolars is by far more debatable: expansion of the dental arch [19], interproximal enamel reduction [20–22], or extraction of one lower incisor [23–25]. Certainly any decision proceeds careful diagnosis. The dentoalveolar discrepancy in the mandible is usually evaluated by measuring, for example, Bolton's ratio, intercanine or intermolar width, lower arch perimeter [26, 27], and last but not least Little's Irregularity Index [28]. The latter not only allows us to quantify the range of crowding [29] but also determines the treatment mode, although its certain drawbacks are obvious. Severe displacement of one or more incisors in the labiolingual direction leads to bias: falsely high values of the discussed index; neglecting tooth-morphology, patients' age, and their facial aesthetics may also be listed. Regardless of all explicit imperfections Little's Irregularity Index has been continuously applied for orthodontic purposes in adults with mandibular incisors crowding [30–32]; therefore an assessment of its current clinical validity seems to be essential in contemporary orthodontics.

## 2. The Aim

Controversy around Little's Irregularity Index and only few studies [33–35] objectively evaluating its power dictated purpose of this study. The aim was both to assess reliability of Little's Irregularity Index 30 years after its introduction and to establish an efficient algorithm for treatment of adults with crowding in the mandibular front area.

## 3. Materials and Methods

**Figure 1:**
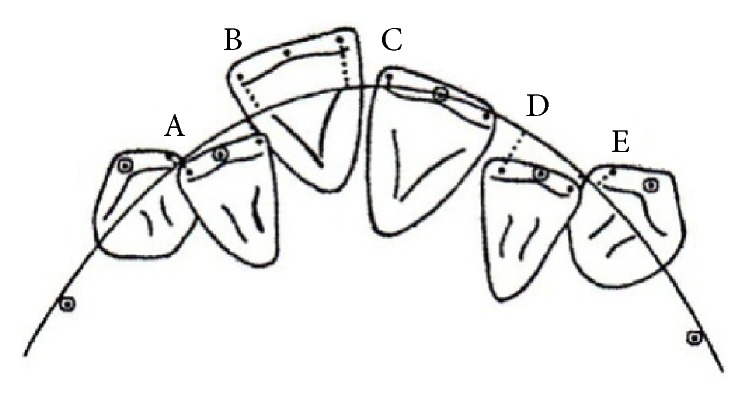
A template applied for measurement Little's Irregularity Index [16]. The sum of linear displacement of anatomical contact points of six mandibular anterior teeth, expressed in mm: 0: perfect alignment, 1–3 mm: minimum irregularity, 4–6 mm: moderate irregularity, 7–9 mm: severe irregularity, and 10 mm and more: very severe irregularity.

Material comprised digital dental casts of 302 patients: 201 women and 101 men, aged from 21 to 39 years, with late crowding of the mandibular anterior teeth prior to treatment (T1). After measuring of Little's Irregularity Index (Figure 1) all patients were allocated into 3 groups (Table 1).

Subsequently Little's Irregularity Index was calculated after debonding (T2) and one year after treatment (T3) when relapse is the most likely to occur.

## 4. Statistical Analysis

The obtained data was analyzed using Statistica software (Statistica 15.0, SPSS, Chicago, IL) utilizing

1. Shapiro-Wilk test to check normality of data distribution and Levene's test to check homogeneity of variance,
2. Student's *t*-test for independent and dependent variables, Wilcoxon's nonparametric tests for dependent variables, or Mann–Whitney's test for independent ones.

The significance level was set at *p* < 0.05.

## 5. Results

**Figure 2:**
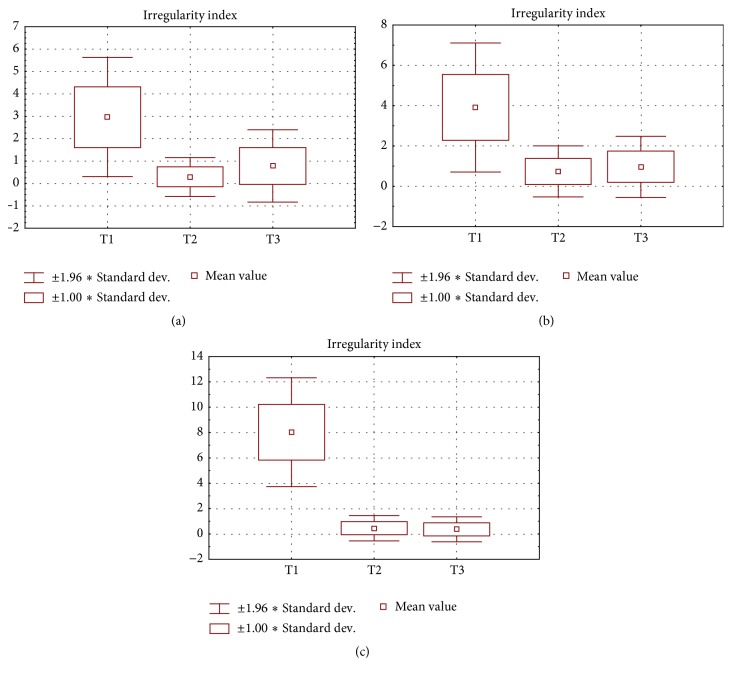
Results of statistic analysis of Little's Irregularity Index values in (a) group 1, (b) group 2, and (c) group 3.

**Figure 3:**
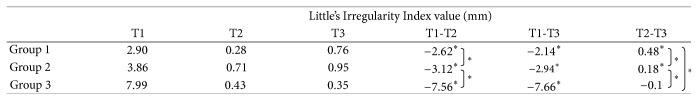
Mean values of Little's Irregularity Index and their changes from beginning (T1) via leveling (T2) until retention stage (T3) of an orthodontic treatment, ^*∗*^*p* < 0.05.

The statistic results of Little's Irregularity Index values and their changes from T1 to T3 are presented in Figures 2 and 3. All values of the discussed index reduced after treatment, in T2 stage. The lowest and the highest values were found in groups 1 and 3, respectively, proving that the dental arch expansion in the properly selected cases is the most efficient treatment method to align the mandibular front teeth. Changes of Little's Irregularity Index values achieved from T1 to T2 were significantly different (*p* < 0.05) in all groups separately; as for intragroup comparison groups 1 and 2 as well as groups 2 and 3 varied significantly (*p* < 0.05).

Final improvement of occlusion, namely, changes of Little's Irregularity Index values achieved with different treatment modalities from T1 to T3, was similarly efficient, which was proved by statistic intragroup evaluation (*p* > 0.05); intergroup assessment of changes of Little's Irregularity Index values turned out to be statistically significant in all cases again proving the efficiency and fully justifying selection of the treatment method.

The most severe relapse of crowding was noted in group 1, where statistically significant (*p* = 0.00) increase of Little's Irregularity Index reached 0.48 mm in the period from T2 to T3. This difference was more than twice smaller in group 2: it equaled 0.18 mm still displaying statistic significance (*p* = 0.02). Only in group 3 Little's Irregularity Index the value decreased by 0.1 mm from T2 to T3 but in statistically insignificant manner (*p* = 0.11) showing the minor improvement of occlusion that occurred during retention stage. At the same time this stability achieved in group 3 was statistically significant (*p* = 0.00) comparing with the changes obtained in patients treated with expansion (group 1) and interproximal enamel reduction (group 2).

## 6. Discussion

Literature reports prove that sticking to specific limits of Little's Irregularity Index values while choosing a method of treatment of the mandibular anterior teeth crowding in adults determines obtaining the successful outcomes. The evidence is brought by case reports where Little's Irregularity Index values were efficiently reduced. Crowding expressed by the index less than 3 mm or ranging from 3 to 4 mm and from 6 to 9 mm was alleviated by dental arch expansion [36–38], interproximal enamel reduction [39–41], and last but not least extraction of the lower incisor [42–44], respectively. That is why following the standards recommended by researchers and in order to obtain the reproducible and reliable treatment results we followed the same algorithm in the current study. We allocated the patients to suitable groups dependent on the treatment mode carefully assessing Little's Irregularity Index values prior to treatment (T1): 2.90 mm before expansion, 3.86 mm before interproximal enamel reduction, and 7.99 mm before extraction of the lower incisor. Clinical results obtained in our study could therefore support the evidence that the limits of values of Little's Irregularity Index, regardless of reports upon its disadvantages, are the diagnostic measurement, which allows selection of appropriate orthodontic biomechanics, namely, treatment option. Obeying those limits leads to stable outcomes that are functionally and aesthetically satisfying when treating adults presenting with dental crowding in the mandible.

Despite Little's Irregularity Index values posttreatment reduction up to 0.28, 0.71, and 0.43 in groups 1 (dental arch expansion), 2 (interproximal stripping), and 3 (extraction of the lower incisor), respectively, none of the patients from study groups presented with the value that dropped to 0 mm in T2 period. Value of the index was closest to an ideal 0 mm in group 1, which indicates susceptibility of the mandibular dental arch to expansion, even in adults. It is in accordance with the results obtained by Pandis [45], Scott et al. [46], and Fleming et al. [47] who efficiently alleviated crowding in mandibular front area by proclining the lower incisors and increase the intercanine width. The highest value recorded in group 2 indicated the least efficient reduction of crowding at T2 stage when interproximal enamel reduction was chosen as a treatment method. It may be partially justified based on the results published by Valli de Almeida et al. [48]. They proved that stripping is efficient only in conservative treatment of teeth with a triangular shape displaying potential for wear, provided that pleasant profile requiring minor changes, Class I, Bolton Index ≤ 3 mm, or mild to moderate mandibular crowding with normal overjet and overbite exist, not to mention low incidence of caries and proper oral hygiene.

Since they also emphasized that the treatment plan should be confirmed by set-up model tests, it becomes apparent that the list of diagnostic indicators leading to interproximal stripping should not be limited to Little's Irregularity Index itself if the interproximal stripping is a method of choice.

Our studies showed no statistically significant intergroup difference in Little's Irregularity Index value changes obtained from the beginning of therapy up to one year of retention (T1–T3) allowing us to conclude that Little's Irregularity Index is a reliable diagnostic tool since similar and expected improvement of occlusion occurs after extremely different protocols of therapy have been applied.

As for the T2 to T3 period statistically significant relapse occurred after expansion (group 1) and after interproximal enamel reduction (group 2): Little's Irregularity Index values were doubled in group 1 comparing with group 2. It is quite likely that unstable enlargement of intercanine width is a cause of such relapse in both groups, more intense after expansion than after interproximal stripping, since the discussed width is irreversibly determined by 9 years of the patient's age [49, 50]. According to Staley et al. [49] an intercanine width value ranging from 24 to 26 mm guarantees stability of alleviation of crowding in the mandible in adult patients. This hypothesis is also confirmed by reports from Glenn et al. [51], Preston [52], and Lee [53] who univocally emphasize that maintaining the intercanine width during orthodontic treatment makes outcomes of the latter stable. Simultaneously only group 3 displayed no relapse: Little's Irregularity Index values improved with time of retention decreasing by 0.1 mm, however, in statistically insignificant manner. Intergroup comparison proved significant difference (*p* < 0.05) of Little's Irregularity Index improvement in group 3 when comparing with group 1 or 2. Groups 1 and 2 also varied significantly (*p* < 0.05). Therefore, it may be concluded that extraction of the lower incisor is most effective in adults with crowding; furthermore, it is stable for at least a year after alleviation. Hegde et al. [54], Barbosa [55], and Valli de Almeida et al. [48] reported similar results as well as Zhylich and Suri [56] who established individual indications for extraction of one of the mandibular incisors. On the contrary, Kahl-Nieke et al. [57] while evaluating postretention crowding and incisor irregularity found that stripping is more stable than extraction of one of the lower incisors in a long-term follow-up evaluation, although in insignificant manner.

Regretfully further feasible comparison of our results with those obtained by other clinicians is impossible due to the lack of original studies present in the literature. Nevertheless, one cannot forget that Little's Irregularity Index values achieved in our study during the retention period did not exceed 0.5 mm in any of the groups, which is why 30 years after the introduction of Little's Irregularity Index it is still effective and reliable clinical indicator of stability provided that the treatment methods based on well-defined limits of the discussed index are chosen.

## 7. Conclusion

Thirty years after its introduction Little's Irregularity Index is a parameter that—provided the appropriate range of values displaying dentoalveolar discrepancy is obeyed—allows selection of reliable treatment method even in adults: (1) up to 3 mm: expansion, (2) from 3 to 5 mm: interproximal enamel reduction, and (3) above 5 mm: extraction; thus relapse may be avoided. However, it should be emphasized that since the most stable results are obtained after extraction of one of the mandibular incisors, that is to say, in cases where Little's Irregularity Index value exceeds 5 mm, thus cases with the lower values should be approached with caution, especially after the growth has been completed.

## Figures and Tables

**Table 1:** 

Group	Dentoalveolar discrepancy	Treatment option	Number (*n*)
1	Less than 3 mm	Expansion of the dental arch	100
2	From 1 to less than 5 mm	Interproximal enamel reduction	101
3	More than 5 mm	Extraction of one lower incisor	101
